# Mapping Problematic Drinking Trends over Time in Urban, Semi-Urban, and Rural Populations

**DOI:** 10.3390/ijerph19010589

**Published:** 2022-01-05

**Authors:** Stefan Bozic, Don Vicendese, Michael Livingston, Bircan Erbas

**Affiliations:** 1 School of Psychology and Public Health, La Trobe University, Melbourne 3083, Australia; stefan.bozic@live.vu.edu.au; 2 The Department of Mathematics and Statistics, La Trobe University, Melbourne 3083, Australia; vicendese.don@gmail.com; 3 The Melbourne School of Population and Global Health, University of Melbourne, Carlton 3053, Australia; 4 Centre for Alcohol Policy Research, School of Psychology and Public Health, La Trobe University, Melbourne 3083, Australia; michael.livingston@curtin.edu.au; 5 National Drug Research Institute, Curtin University, Perth 6845, Australia

**Keywords:** alcohol, urban, semi-urban, trends, long-term risky drinking, heavy-episodic drinking, mental health

## Abstract

Current alcohol public health policy in Australia is not uniform but is generally focused on restricting access and early prevention of problematic alcohol use. Semi-urban and rural populations are at greater risk of disease and other poor health outcomes due to a variety of factors. Little is known about problematic drinking patterns over time in semi-urban and rural populations. This study aims to assess patterns of problematic drinking defined as both long-term risky and heavy episodic drinking over time by age, sex, and mental health status among urban, semi-urban and rural populations). Four waves (2004 to 2016) of the Australian NDSHS (National Drug Strategy Household Survey) were analyzed to assess problematic drinking of participants over 18 years of age. We used regression models and predictive margins to identify trends in problematic drinking over time based on age, sex, and mental health status. Our results show young adults across all regions, males, and mentally well individuals in urban areas have reductions in the risk of problematic drinking over time. Middle-aged adults across all regions, females, and those with varying mental health presentations in rural areas have some increases in risk of problematic drinking over time. The general conclusion is that targeted alcohol-related public health policy may need to change and focus on females, middle-aged individuals, and those living in rural areas. Programs to support problematic drinking in people with mental health disorders may also need to be a priority.

## 1. Introduction

Alcohol consumption in the 21st century is a multifaceted issue that poses social, psychological, health, and anthropological problems [1]. Although alcohol plays an important role in Australian identity and culture [2], its misuse contributes to widespread harm. Drinking to excess means individuals are increasing their risk of alcohol poisoning, severe mental health episodes, including alcohol-induced psychosis and suicide, and death as a result of risk-taking behavior, including high-risk driving [3,4].

Changes in alcohol consumption and cultural attitudes towards consumption have changed over time. Increased wealth in both developed and developing countries contributes to increased alcohol consumption [5,6], in addition to acculturation-related factors for long-term migrants and first-generation descendants [7,8]. Deregulation of alcohol sales over time result in increased alcohol outlets and hours of operation in low socio-economic status (SES) neighborhoods [9]. Subsequently, these patterns of increased alcohol use lead to problematic drinking and the negative effects associated with it [10].

In Australia, reported time trends indicated that per capita consumption of high-risk (defined as 2 standard drinks or more on average per day) drinkers increased from 20.7 L in 2001 to 21.5 L [11]. In some instances, time trends indicated that females aged 45–54 were a subgroup of the population that demonstrated increases in alcohol intake [12]. Studies in Europe and Asia have compared alcohol trends in rural and urban populations. In Belarus between 1990 and 2005 and over a four-wave period, it was reported that alcohol-poisoning rates rose steadily in all location groups, with rural dwellers being impacted the worst. Due to the struggling Belarusian economy and the isolation of rural villages in Belarus, the difference in alcohol-poisoning rates (per 100,000) between urban and rural populations jumped steadily from 37% in 1990 to 50% in 2005 [13]. Amongst the rural Thai population, alcohol-related harm increased from 24.0% in 2007 to 28.1% in 2017 [14]. Several Australian studies reported that rural Australians experience higher rates of alcohol abuse compared to urban counterparts. Males living in rural settings are significantly more likely than their urban counterparts to consume alcohol daily (4% greater difference in risk) and drinking in excess (8% greater risk difference) [15]. No Australian study has assessed these specific patterns over time.

Some studies have assessed problematic drinking time trends by age and sex. Problematic drinking peaks between the ages of 24–26, a phenomenon that is more prominent amongst males than females [16]. Long-term risky drinking across the UK, Australia, and South Africa show distinct trends amongst males with peaks occurring in the middle-age categories while heavy episodic drinking peaks earlier [17]. In the U.S. between the years 2002 and 2017, females (18–44) were the only group of people to increase their prevalence of heavy episodic drinking (1.7%) when data were analyzed over time and not cross-sectionally [18].

The complexities of alcohol dependence become aggravated when one has a mental illness. When an individual has both a mental health issue and substance use issue, this condition is known as a dual diagnosis [19]. Although dual diagnosis is a condition that incorporates two major public health issues, its understanding is lacking and is generally only studied in United States population groups [20,21]. In our study, we reported that psychological distress had a significant impact on increased risk for problematic alcohol use in females only [22].

We know little of the associations between mental health outcomes and problematic drinking trends over time in semi-urban and rural populations. Given these knowledge gaps, we sought to examine time trends of problematic drinking outcomes in urban, semi-urban, and rural areas using the Australian National Drug Strategy Household Survey (NDSHS). In particular, we aimed to both examine trends in alcohol-related use (heavy episodic drinking and long-term risky drinking) for sex, age groups based on location, and assess comorbidity trends for mental health status and alcohol-related use (heavy episodic drinking and long-term risky drinking) based on location and over time.

## 2. Materials and Methods

### 2.1. Design

The present study used five waves of the cross-sectional NDSHS (from 2004 to 2016), an Australia-wide health survey that collects self-reported drug and alcohol data from different sub-population groups. Overall, a total of 124,597 participants aged between 14 and 99 years of age (*n* = 55,280 males and *n* = 69,317 female) were included. Ethical approval was granted from the La Trobe university human ethics committee (HREC #: 19124).

### 2.2. Outcome Variables

Problematic drinking status was the primary dependent variable for this study and was measured by a standalone question:

*Please record how often in the last 12 months you have had each of the following number of standard drinks in a day?*

In total, there are 64 combinations that can be chosen and measure both volume and frequency of drinking. For volume, there are right responses that range from none to 20 or more standard drinks a day. For frequency, there are eight responses ranging from never to every day. The total annual consumption is derived by multiplying the mid-point of the volume and frequency category. Based on the responses, a response was categorized as either two forms of problematic drinking. Engaging in heavy episodic risky drinking was determined by the consumption of 5 or more standard drinks in one sitting at least once a month and defined as “heavy episodic drinking”. Engaging in long-term risky drinking was determined by the consumption of 2 or more standard drinks per day over a 12-month period and defined as “long-term risky drinking”. These thresholds are taken from the National Health and Medical Research Council’s low-risk drinking guidelines [23].

### 2.3. Other Variables

We considered variables for psychological well-being, location, age and sex for inclusion. For psychological well-being, the NDSHS utilizes the Kessler Psychological Distress Scale (K10) to determine the level of psychological well-being. The K10 is a brief screening scale for non-specific psychological distress. However, the focus of the scale is on anxiety and depression. It has been used in many Australian population health surveys since the late 1990s. Detailed descriptions of the K10 can be found in the relevant article [24]. Each question is scored with the lowest (none of the time) being worth one point and (all of the time) being worth five points [25]. Participants can score between 10 and 50 on this scale, with higher scores representing more psychological distress. We classified participants into one of four states:

(a) Likely to be well (10–15)
(b) Likely to have a mild mental disorder (16–21)
(c) Likely to have a moderate mental disorder (22–29)
(d) Likely to have a severe mental disorder (30–50)

An open-ended and standalone question was used to capture age (in years). Based on these, participants were grouped into the following age groups: (18–24), (25–29), (30–39), (40–49), (50–59), (60–69) and 70+. A standalone question requested one’s postcode, which was matched with its region and coded the participant as either living in an urban, semi-urban, or rural area.

### 2.4. Statistical Analysis

Data are presented as proportions for each wave (2004 to 2016). For each outcome of long-term risky and heavy episodic drinking, we used logistic regression methods. In each regression model, we assessed associations between sex, age, and psychological well-being stratified by wave. For each outcome, separate models were fitted for urban, semi-urban, and rural populations. Study year was interacted with these variables to assess change over time. Pairwise comparisons were made for each year and time trends were statistically assessed using trend analyses based on predicted marginal change (displayed in graphical form). Statistical significance was set at 0.05. The results are presented as odds ratios (OR) and 95% confidence intervals (Cis). Statistical analyses were performed using Stata release 14.1 [26].

## 3. Results

### 3.1. Population Characteristics

Characteristics of the population are displayed in Table S1 (online Supplement Table S1). Middle-aged groups made up a sizable proportion of samples within each year. Most of the population identified as mentally well. Both the proportion of heavy episodic drinking and long-term risky drinking appeared to decrease over time. Subjects came from all of the Australian region groups including: urban (*n* = 80,458), semi-urban areas (*n* = 25,053)) and also, remote areas (*n* = 19,058).

### 3.2. Overall Problematic Drinking Trends over Time by Location

Time-trend figures are displayed as online supplements.

#### 3.2.1. Heavy Episodic Drinking

**Figure 1:**
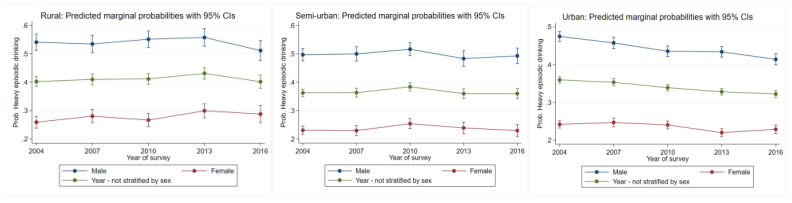
Change over study years for heavy episodic drinking marginal probabilities for urban, semi-urban, and rural dwellers by sex. Each graph also has overall changes over time not stratified by the variable of interest.

Between 2004 and 2016, there was a consistent reduction in the likelihood to engage in heavy episodic drinking in urban areas. Between 2004 and 2010, there was a significant increase in risk in semi-urban areas (*p* = 0.03, Figure 1). In rural areas, between 2004 and 2013, there was a significant increase in risk (*p* = 0.04), but this trend reduced to 2004 levels thereafter.

#### 3.2.2. Long-Term Risky Drinking

**Figure 2:**
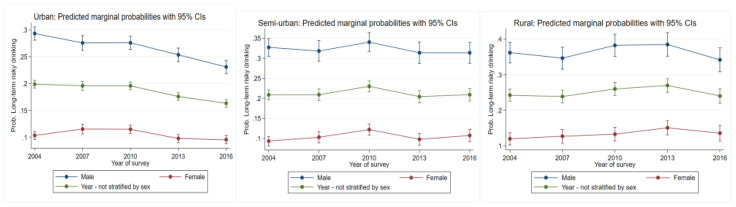
Change over study years for long-term risky drinking marginal probabilities for urban, semi-urban, and rural dwellers by sex. Each graph also has overall change over time not stratified by the variable of interest.

In urban areas, there was a consistent reduction in the likelihood to engage in long-term risky drinking over time (*p* = 0.000, Figure 2). For semi-urban areas, there was a significant increase in risk up to 2010 (*p* = 0.003) and then returned to 2004 levels. In rural areas, an increased risk up to 2013 occurred, with a reduction to 2004 levels (*p* = 0.01).

### 3.3. Problematic Drinking Trends over Time by Sex

#### 3.3.1. Heavy Episodic Drinking

For males residing in urban areas, there was a consistent reduction in the likelihood to engage in heavy episodic drinking over time (*p* = 0.000, Figure 1, Table 1). For females, the likelihood to engage in heavy episodic drinking decreased between 2010 and 2013 (*p* = 0.007). For semi-urban areas, males were at greater risk overall than females. Despite some oscillations in risk between the study years for both females and males, there was neither a statistically significant change nor a statistically significant trend over time. The likelihood to engage in heavy episodic drinking over time significantly increased for females residing in rural areas up to 2013 (*p* = 0.02). Despite oscillating risk for men, there were non-significant between the study years.

#### 3.3.2. Long-Term Risky Drinking

In all areas, males were at a greater risk overall than females. For males, in urban areas there was a consistent reduction in the likelihood to engage in long-term risky drinking (*p* = 0.000, Figure 2, Table 2). For females, risk increased up to 2010 (*p* = 0.04) and declined thereafter. Semi-urban areas females had significant increases in risk up to 2010 (*p* = 0.002), followed by a decline in 2013 and a slight increase thereafter. Risk for both males and females residing in rural areas were similar over time. Despite oscillating risk for males between 2004 and 2016, these proved to be non-significant.

### 3.4. Problematic Drinking Trends over Time by Age Group

#### 3.4.1. Heavy Episodic Drinking

**Figure 3:**
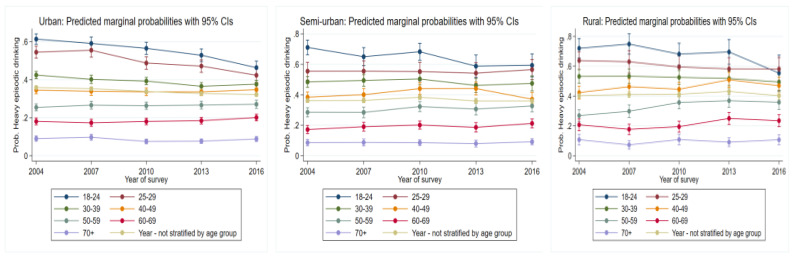
Year-old group (*p* = 0.01). Risk for the 40–49 group significantly increased up to 2013 but later declined (*p* = 0.03). The 60–69-year age group were at an increased risk of heavy episodic drinking over time (*p* = 0.04).

For those living in urban areas, over time, the 18–24-, 25–29, and 30–39-year-old groups (*p* = 0.000, Figure 3, Table 1) all had consistent reductions in risk of heavy episodic drinking. In contrast, the 60–69-year age group consistently demonstrated increases in risk over time (*p* = 0.03).

In rural areas, there was a consistent reduction in risk of heavy episodic drinking for the 18–24-year age group (*p* = 0.01). Between 2004 and 2013, the 40–49-year-old group had significant increases in risk (*p* = 0.01). By 2016, this increase had leveled out. The 50–59-year-old group had a consistent increase in risk over time (*p* = 0.000). The risk for heavy risky drinking remained relatively unchanged for the 60–69-year-old group between 2004 and 2007. However, between 2007 and 2016, there was a significant increase in risk (*p* = 0.03).

#### 3.4.2. Long-Term Risky Drinking

**Figure 4:**
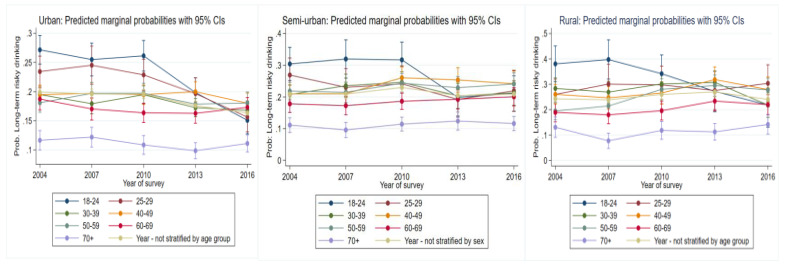
Change over study years for long-term risky drinking marginal probabilities for urban, semi-urban, and rural dwellers by age group.

For those living in urban areas, most age groups displayed a reduction in risk of long-term risky drinking behaviors (Figure 4, Table 2). Despite a non-significant increase between 2004 and 2007, there was a significant decrease in risk for the 70+ age group between 2007 and 2013 (*p* = 0.04). Although it returned to 2004 levels, this increase was non-significant.

For semi-urban areas, the likelihood to engage in long-term risky drinking for the 18–24-year-old group remained steady until 2010, before there was a significant reduction thereafter (*p* = 0.02). For the 40–49-year-old group, risk had increased up to 2010 (*p* = 0.03) but leveled out thereafter. Despite oscillating risk being evident in the other age groups, these remained non-significant. In rural areas, over time, there was a consistent reduction in risk for the 18 to 24 age groups and 30–39 age groups (*p* = 0.001, *p* = 0.04), respectively. Between 2007 and 2013, there was a significant increase in risk in the 40–49-year age group (*p* = 0.04). Similarly, there was a consistent increase in risk for the 50–59-year-old group (*p* = 0.04). Between 2007 and 2013, there was a significant increase in risk for the 60–69-year age group (*p* = 0.04), but by 2016, these increases had leveled out. Between 2007 and 2016 there was a significant increase in risk for the 70 + year age group (*p* = 0.01).

### 3.5. Problematic Drinking Trends over Time-Based on Psychological Well-Being

#### 3.5.1. Heavy Episodic Drinking

**Figure 5:**
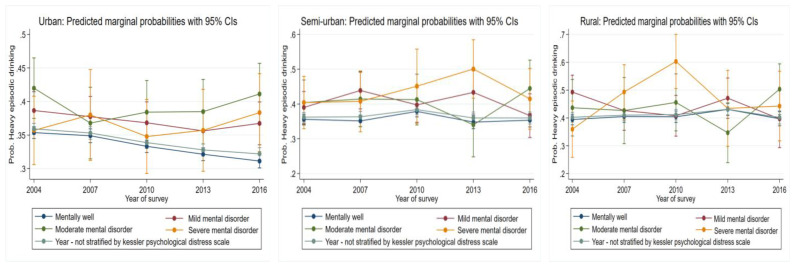
Change over study years for heavy-episodic drinking marginal probabilities for urban, semi-urban, and rural dwellers by psychological well-being status.

Overall, from urban areas, those reporting both mild and moderate mental well-being were associated with an increased likelihood of heavy episodic drinking. Over time, there was a consistent reduction in the likelihood to engage in heavy episodic drinking for those who were psychologically well (*p* = 0.000, Figure 5, Table 1). Despite oscillating risk for those likely to have mild, moderate, and severe mental illnesses, these changes were non-significant. Risk of heavy episodic drinking among semi-urban areas and those who were mentally well increased up to 2010 (*p* = 0.01) then declined thereafter (*p* = 0.01). Despite oscillating risk between 2004 and 2016 for those who were likely to be mildly, moderately, and severely mentally ill, these changes remained non-significant. In rural areas, for those who reported being mentally well, risk increased significantly up to 2013 (*p* = 0.01) and then declined thereafter. For those likely to have a moderate mental disorder, there was a significant and substantial increase in risk between 2013 and 2016 (*p* = 0.03). Significant and substantial increases in risk were also identified for those likely to have a severe mental illness up to 2010 (*p* = 0.001), which declined thereafter.

#### 3.5.2. Long-Term Risky Drinking

**Figure 6:**
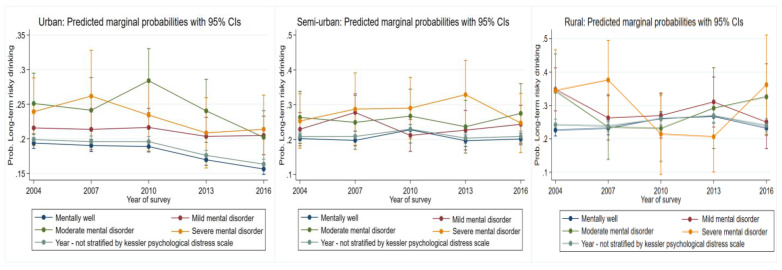
Change over study years for long-term risky drinking marginal probabilities for urban, semi-urban, and rural dwellers by psychological well-being status.

There was a consistent reduction in risk of long-term risky drinking for those who were mentally well residing in urban areas (*p* = 0.000, Figure 6, Table 2). Risk for those likely to have a moderate, mild, and severe mental illness was oscillating over time. However, these were non-significant. For semi-urban areas, risk for those who were mentally well were significant up to 2010 (*p* = 0.01) but declined thereafter. Despite oscillating risk for those who were likely to have a mild, moderately, and severely mentally ill, these remained non-significant. For those residing in rural areas, risk of long-term risky drinking significantly increased for those who were mentally well (*p* = 0.003), but by 2016, these increases had leveled out. Despite oscillating risk for those likely to have a mild and moderate mental disorder, these were non-significant. The risk for those likely to have a severe mental disorder declined between 2007 and 2013.

## 4. Discussion

Our study suggests differences in problematic drinking behaviors over time among urban, semi-urban and rural areas. Patterns exist with problematic drinking to varying degrees based on age group, psychological well-being and sex.

Our findings are consistent with others identifying middle-aged females as an emerging at-risk group as they are not reducing their drinking over time [12,18,27]. Although the time trends for females, in general, had an oscillating risk, increasing risk was present over time in rural areas. Norwegian data from 1994 to 2016 shows a narrowing of the gender gap for problematic alcohol use [28]. As rural drinking in Australia is seen as the personification of male traits [29], intersectional feminist theory would argue that growing alcohol consumption amongst women is a result of females rejecting collective female norms [30]. Adopting these historically male traits, coupled with greater alcohol sponsorship of rural Australian sports clubs [31] and convenient accessibility of alcohol in rural areas [32], could be impacting on rural female alcohol consumption.

Middle-aged groups, especially those residing in rural areas also had increasing risk of problematic drinking over time. The middle-aged group, known as baby boomers, failing to reduce problematic drinking over time is consistent with other Australian time-trend data [33]. As the baby boomer cohort consumes most of their alcohol in the home [33], they in turn avoid responsible service of alcohol regulations that periodically reduce one’s ability to engage in problematic drinking in venues [34]. Alcohol-related policy and health promotion campaigns could shift their focus and begin to target these “at-risk” groups, including females and middle-aged adult populations.

Our findings highlight decreasing risk of problematic drinking over time for those who are mentally well in urban areas. Further out from the urban area, mentally well and also those likely to be moderately mentally ill had increased risk in problematic drinking over time. Our results suggest these associations with problematic drinking appear to become prominent for those who are likely to be moderately mentally ill, especially for heavy episodic drinking in rural areas. More studies are needed to replicate our findings as few have assessed these associations over time. In terms of mental health, rural life is markedly different from metropolitan life in a number of areas including: susceptibility to severe economic hardship isolation and poor job opportunities [35]. This coupled with limited access to mental health services over time in rural Australia [36] could potentially explain the observed associations between poor psychological well-being and problematic alcohol use over time.

Previous research has mainly been cross-sectional, with only a small amount of trend research in this field [37]. Although our results were generally non-significant over time, significant results in rural areas for females, people with varying psychological distress, and middle-aged individuals may be due to the rapid population changes occurring in Australia. Australia has seen an unplanned yet massive expansion of suburban environments in major cities. Despite the majority of Australian capital cities being monocentric, the trend from 2001 to 2016 has been greater migration flows to outer suburbs, away from urban capital centers [38].

Although the issues are complex, more studies are needed to focus on the environmental and behavioral determinants of these trends to enable a better understanding of the contribution of socially disadvantaged, varying ethnicities, and culturally diverse communities on these ever-changing trends over time.

Problematic drinking is not only an individual burden but also a burden on extended family, the community, local and national economies [39,40]. Past research has shown that these burdens are not straightforward and stem from different population groups based on location and individual differences such as gender and age. The interconnected world we live in and the advancement of globalization is creating both population, demographic and social determinants of health changes that are becoming increasingly complex [41]. By assessing these trends epidemiologically, we can then inform policy revisions to target at-risk populations for problematic drinking in order to help mitigate the impact it has at an individual, community, and national level.

We have a number of strengths in our study. The large sample size at the different time periods enabled sufficient power to detect multiple interactions between key variables. However, limitations of the data source can question this robustness and should be considered when making future inferences, given the lack of data to suggest causality. It is worth noting the limitation of social desirability bias, the inclination to describe one in a more positive light. People who consider themselves to be healthy are generally more likely to respond to a health survey than those who have a chronic condition; respondents with a chronic condition either ignore the survey or respond in a positive way [42]. Additionally, the results of this study stem from pre 2019/2020 when the COVID-19 pandemic had a substantive impact on problematic drinking [43].

## 5. Conclusions

In summary, our findings suggest that from the period of 2004 to 2016, females, middle-aged individuals, and those with varying psychological well-being presentations in rural areas are emerging at-risk groups of problematic drinking. More studies are needed to replicate our findings in order to contribute to the development of targeted interventions to reduce this increasing risk.

## Figures and Tables

**Table 1:** Heavy episodic drinking logistic regression models for urban, semi-urban and rural dwellers.

Urban N = 80,458	Heavy Episodic Drinking		Year			
			2007	2010	2013	2016
Main Effects OR (CI) for Covariates Below			Covariate X Year Interaction OR (CI)			
**Sex** **(Ref-Male)**	Female	0.30(0.28–0.33)	1.13(0.99–1.30)	1.22(1.08–1.39)	1.11(0.97–1.26)	1.31(1.15–1.49)
**Age group** **(Ref-18–24)**	25–29	0.73(0.61–0.88)	1.17(0.87–1.55)	0.97(0.75–1.28)	1.07(0.81–1.40)	1.15(0.86–1.52)
	30–39	0.43(0.37–0.50)	1.02(0.80–1.29)	1.10(0.88–1.38)	1.13(0.90–1.42)	1.58(1.26–2.00)
	40–49	0.30(0.26–0.35)	1.10(0.86–1.40)	1.22(0.97–1.54)	1.42(1.12–1.80)	2.00(1.58–2.53)
	50–59	0.19(0.16–0.22)	1.22(0.95–1.56)	1.36(1.07–1.72)	1.59(1.24–2.03)	2.19(1.71–2.81)
	60–69	0.12(0.10–0.14)	1.09(0.83–1.43)	1.30(1.00–1.68)	1.53(1.78–1.99)	2.31(1.78–3.00)
	70+	0.06(0.04–0.06)	1.25(0.90–1.72)	1.07(0.78–1.47)	1.25(0.91–1.71)	1.98(1.45–2.71)
**Kessler** **(Ref–Well)**	Mild	1.19(1.02–1.39)	0.97(0.78–1.22)	1.01(0.80–1.26)	1.10(0.80–1.26)	1.11(0.89–1.39)
	Moderate	1.41(1.12–1.78)	0.78(0.54–1.13)	0.92(0.66–1.29)	0.98(0.70–1.38)	1.15(0.84–1.59)
	Severe	1.02(0.77–1.34)	1.15(0.74–1.80)	1.06(0.71–1.58)	1.18(0.78–1.80)	1.40(0.95–2.08)
**Semi-Urban** **N = 25,053**						
**Sex** **(Ref-Male)**	Female	0.23(0.20–0.27)	1.01(0.89–1.28)	1.10 (0.88–1.37)	1.21 (0.95–1.54)	1.11(0.87–1.42)
**Age group** **(Ref-18–24)**	25–29	0.47(0.32–0.68)	1.37(0.78–2.41)	1.16 (0.67–2.00)	1.74(0.97–3.14)	1.89(1.05–3.41)
	30–39	0.34(0.25–0.46)	1.43(0.90–2.27)	1.28 (0.82–2.02)	1.68(1.02–2.77)	1.74(1.04–2.90)
	40–49	0.21(0.16–0.29)	1.47(0.92–2.36)	1.53(0.97–2.42)	2.44(1.49–4.00)	1.71(1.02–2.86)
	50–59	0.13(0.09–0.18)	1.36(0.85–2.19)	1.45(0.92–2.29)	2.13(1.29–3.52)	2.28(1.37–3.79)
	60–69	0.06(0.05–0.09)	1.57(0.95–2.57)	1.48(0.92–2.38)	2.14(1.26–3.62)	2.44(1.45–4.13)
	70+	0.03(0.02–0.04)	1.39(0.78–2.47)	1.20(0.69–2.08)	1.78(0.97–3.28)	1.99(1.11–3.58)
**Kessler** **(Ref–Well)**	Mild	1.12(0.93–1.58)	1.33(0.89–1.98)	0.91(0.62–1.35)	1.29(0.84–1.98)	0.89(0.57–1.38)
	Moderate	1.31(0.91–1.90)	1.08(0.61–1.90)	0.92(0.53–1.57)	0.73(0.39–1.38)	1.23(0.70–2.15)
	Severe	1.31(0.86–2.00)	1.04(0.55–1.97)	1.12(0.55–2.27)	1.68(0.91–3.09)	1.05(0.56–1.97)
**Rural** **N = 19,058**						
**Sex** **(Ref-Male)**	Female	0.23(0.18–0.28)	1.15(0.85–1.56)	1.05(0.78–1.41)	1.26(0.94–1.69)	1.54(1.13–2.09)
**Age group** **(Ref-18–24)**	25–29	0.65(0.41–1.04)	0.83(0.41–1.67)	1.00(0.51–2.00)	0.89(0.43–1.82)	1.72(0.79–3.74)
	30–39	0.40(0.26–0.60)	0.88(0.48–1.62)	1.20(0.66–2.19)	1.10(0.59–2.08)	1.95(0.96–3.97)
	40–49	0.24(0.16–0.37)	1.05(0.57–1.97)	1.38(0.75–2.52)	1.75(0.92–3.32)	2.89(1.42–5.88)
	50–59	0.11(0.07–0.17)	1.05(0.56–1.98)	1.97(1.07–3.64)	2.03(1.06–3.88)	3.85(1.88–7.87)
	60–69	0.08(0.05–0.12)	0.73(0.38–1.42)	1.14(0.60–2.19)	1.60(0.82–3.14)	2.96(1.42–6.16)
	70+	0.03(0.02–0.06)	0.58(0.26–1.32)	1.26(0.58–2.72)	1.03(0.47–2.27)	2.54(1.11–5.86)
**Kessler** **(Ref–Well)**	Mild	1.70(1.21–2.39)	0.66(0.39–1.12)	0.60(0.35–1.02)	0.72(0.43–1.20)	0.59(0.32–1.09)
	Moderate	1.26(0.72–2.20)	0.89(0.38–2.11)	1.04(0.48–2.26)	0.51(0.23–1.16)	1.32(0.65–2.70)
	Severe	0.82(0.46–1.48)	1.96(0.88–4.34)	3.43(1.55–7.61)	1.24(0.50–3.08)	1.51(0.65–3.54)

**Table 2:** Long-term risky drinking logistic regression models for urban, semi-urban and rural dwellers.

Urban N = 80,458	Long-Term Risky Drinking		Year			
			2007	2010	2013	2016
Main Effects OR (CI) for Covariates			Covariate X Year Interaction OR (CI)			
**Sex** **(Ref-Male)**	Female	0.27(0.25–0.30)	**1.24(1.06–1.44)**	1.23(1.06–1.41)	**1.16(1.00–1.34)**	1.28(1.10–1.49)
**Age group** **(Ref-18–24)**	25–29	0.81(0.66–0.99)	**1.17(0.85–1.61)**	1.03(0.76–1.38)	**1.22(0.89–1.69)**	1.29(0.91–1.82)
	30–39	0.63(0.53–0.75)	**0.99(0.76–1.29)**	1.06(0.83–1.36)	**1.33(1.02–1.74)**	1.81(1.36–2.41)
	40–49	0.63(0.53–0.75)	**1.12(0.86–1.46)**	1.07(0.83–1.37)	**1.61(1.23–2.10)**	1.98(1.49–2.64)
	50–59	0.57(0.48–0.68)	**1.23(0.94–1.61)**	1.91(0.92–1.54)	**1.53(1.16–2.02)**	2.19(1.63–2.93)
	60–69	0.60(0.50–0.72)	**0.98(0.74–1.30)**	0.90(0.69–1.17)	**1.30(0.98–1.73)**	1.98(1.48–2.66)
	70+	0.33(0.27–0.41)	**1.17(0.86–1.60)**	0.90(0.72–1.35)	**1.29(0.94–1.77)**	2.09(1.51–2.90)
**Kessler** **(Ref–Well)**	Mild	1.16(0.97–1.37)	**1.01(0.77–1.31)**	1.04(0.81–1.33)	**1.10(0.85–1.42)**	1.22(0.94–1.58)
	Moderate	1.43(1.10–1.86)	**0.96(0.66–1.41)**	1.23(0.86–1.77)	**1.11(0.76–1.62)**	0.97(0.67–1.40)
	Severe	1.34(0.99–1.80)	**1.16(0.72–1.86)**	1.00(0.65–1.54)	**0.98(0.63–1.53)**	1.12(0.72–1.72)
**Semi Urban** **N = 25,053**						
**Sex** **(Ref-Male)**	Female	0.20(1.17–0.24)	**1.15(0.88–1.50)**	1.28(1.00–1.64)	**1.14(0.86–1.50)**	1.26(0.97–1.65)
**Age group** **(Ref-18–24)**	25–29	0.83(0.55–1.25)	**0.74(0.93–1.38)**	0.80(0.44–1.47)	**1.16(0.58–2.31)**	1.28(0.65–2.52)
	30–39	0.57(0.40–0.80)	**1.10(0.66–1.85)**	1.20(0.74–1.96)	**1.82(1.01–3.28)**	1.79(1.02–3.11)
	40–49	0.57(0.41–0.81)	**0.93(0.56–1.56)**	1.29(0.80–2.08)	**2.45(1.39–4.33)**	2.10(1.21–3.63)
	50–59	0.61(0.44–0.85)	**0.91(0.55–1.49)**	1.10(0.69–1.77)	**2.00(1.14–3.52)**	1.98(1.12–3.37)
	60–69	0.46(0.33–0.65)	**0.89(0.53–1.49)**	1.01(0.62–1.64)	**2.08(1.18–3.67)**	2.03(1.18–3.45)
	70+	0.26(0.18–0.37)	**0.78(0.44–1.38)**	0.99(0.59–1.69)	**2.15(1.16–3.97)**	1.84(1.04–3.26)
**Kessler** **(Ref–Well)**	Mild	1.19(0.97–1.64)	**1.38(0.88–2.17)**	0.76(0.48–1.18)	**1.02(0.63–1.67)**	1.10(0.69–1.74)
	Moderate	1.47(0.94–2.30)	**0.95(0.49–1.83)**	0.59(0.46–1.62)	**0.88(0.46–1.70)**	1.06(0.55–2.06)
	Severe	1.38(0.85–2.23)	**1.26(0.59–2.69)**	1.04(0.52–2.06)	**1.56(0.77–3.18)**	0.97(0.48–1.95)
**Rural** **N = 19,058**						
**Sex** **(Ref-Male)**	Female	0.22(0.18–0.28)	**1.15(0.84–1.57)**	1.06(0.78–1.45)	**1.23(0.90–1.66)**	1.32(0.95–1.82)
**Age group** **(Ref-18–24)**	25–29	0.53(0.33–0.86)	**1.17(0.56–2.42)**	1.49(0.73–3.08)	**1.91(0.89–4.10)**	3.02(1.34–6.85)
	30–39	0.61(0.41–0.91)	**0.86(0.48–1.56)**	1.34(0.74–2.42)	**1.98(1.04–3.74)**	1.64(0.78–3.45)
	40–49	0.53(0.36–0.80)	**0.87(0.47–1.58)**	1.26(0.69–2.29)	**2.38(1.25–4.55)**	2.66(1.29–5.50)
	50–59	0.35(0.23–0.54)	**1.07(0.59–1.96)**	2.06(1.13–3.75)	**3.19(1.68–6.06)**	3.98(1.93–8.22)
	60–69	0.34(0.22–0.52)	**0.88(0.47–1.64)**	1.28(0.69–2.39)	**2.36(1.23–4.54)**	2.97(1.43–6.18)
	70+	0.21(0.13–0.35)	**0.52(0.24–1.11)**	1.09(0.54–2.25)	**1.50(1.23–4.54)**	2.70(1.20–6.07)
**Kessler** **(Ref–Well)**	Mild	1.99(1.40–2.82)	**0.60(0.35–1.04)**	0.53(0.31–0.90)	**0.64(0.37–1.08)**	0.56(0.31–1.01)
	Moderate	1.93(1.07–3.49)	**0.53(0.23–1.24)**	0.43(0.18–1.02)	**0.59(0.24–1.43)**	0.86(0.39–1.87)
	Severe	1.95(1.03–3.70)	**1.13(0.47–2.71)**	0.38(0.14–1.07)	**0.35(0.14–0.92)**	1.01(0.39–2.66)

## Data Availability

Restrictions apply to the availability of these data. Data was obtained from the Australian Data Archive (ADA) and can be obtained directly through the ADA at https://dataverse.ada.edu.au/dataverse.xhtml?alias=ndshs (accessed on 10 November 2021).

## Supplementary Materials

The following supporting information can be downloaded at: https://www.mdpi.com/article/10.3390/ijerph19010589/s1, Table S1: Characteristics of the participants NDSHS (2004–2016).
