# Supplementary material for: Mapping Problematic Drinking Trends over Time in Urban, Semi-Urban, and Rural Populations
Source: Int J Environ Res Public Health. 2022 Jan 5;19(1):589. doi: 10.3390/ijerph19010589 (PMC8744560; doi:10.3390/ijerph19010589)
Supplement: Supplementary file 1 [file ijerph-19-00589-s001.zip › ijerph-1502413-supplementary.pdf]

Table S1. Characteristics of the participants NDSHS (2004–2016).

	2004	2007	2010	2013	2016
N	28,582	22,912	26,157	23,521	23,425
	**Weighted % CI ***	**Weighted % CI ***	**Weighted % CI ***	**Weighted % CI ***	**Weighted %CI ***
**Sex**					
**Male**	49.2	49.4	49.5	49.5	49.4
**Female**	50.8	50.7	50.5	50.5	50.6
**Age groups (years)**					
**18–24**	12.6	12.6	12.6	12.0	11.8
**25–29**	7.8	7.6	8.6	9.0	8.7
**30–39**	18.0	17.4	17.3	16.8	17.1
**40–49**	18.1	17.6	17.2	16.9	16.3
**50–59**	15.4	15.5	15.2	15.5	15.2
**60–69**	11.8	13.0	11.3	12.0	12.6
**70+**	9.6	9.7	11.3	11.8	12.6
**Mental Health Status**					
**Well**	84.9	85.4	85.2	84.9	83.0
**Mild**	9.0	8.8	8.8	8.3	9.0
**Moderate**	3.7	3.6	3.5	3.7	4.5
**Severe**	2.4	2.2	2.6	3.1	3.5
**Heavy Episodic Drinking: ****					
**No**	63.6	64.4	65.1	67.1	68.2
**Yes**	36.4	35.6	34.9	32.9	31.8
**Long-term Risky Drinking: *****					
**No**	80.2	80.5	80.1	82.0	83.1
**Yes**	19.8	19.5	20.0	18.0	17.0
**Location**					
**Urban**	67.6	67.7	68.4	71.2	70.0
**Semi-urban**	21.0	20.4	20.5	17.0	18.9
**Rural**	11.5	11.9	11.1	11.8	11.0

Notes: * Sample counts are unweighted and all proportions are based on weighted data. ** Heavy episodic drinking is characterised as having 5+ standard drinks on an occasion in the last 12 months. *** Long-term risky drinking is characterised as having 2+ standard drinks per day in the last 12 months
